# The progress of South Korean blood transfusion services (2004-2006)

**DOI:** 10.4103/0973-6247.42697

**Published:** 2008-07

**Authors:** Deok Ja Oh

**Affiliations:** *Director of Blood Planning Division, Blood Services Headquarters, Korean Red Cross, Choongku, Seoul-100043*

## The Organization of KRC Blood Services

The Korean Red Cross (KRC) Blood services started in 1957. In 1981 the government entrusted the Blood services to the KRC. The KRC Blood services have a working relationship with government bodies, medical blood banks, and private blood center as a components of blood service in Korea. The 16 blood centers, a blood transfusion research institute, 3 NAT laboratories, a fractionation center are under the management of KRC Blood Service Headquarters. There are three Divisions in the Blood Services Headquarters. Under those, 15 teams are in operation. The three NAT centers established in 2004 are conducting HIV and HCV RNA tests. BIMS (integrated blood information management system) which is implemented since 2000 and which is connected to systems of government side, hospital blood banks, and private blood center through BISS (blood information sharing system). BISS can submit programs such as blood ordering, inquiry of donor eligibility, transmission of information between KRC and other bodies.

## The Statistics of KRC Blood Services

### Blood collection and supply

**Figure 1:**
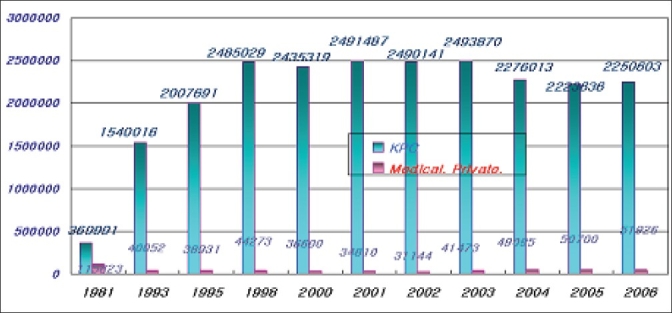
Number of blood donation

**Figure 2:**
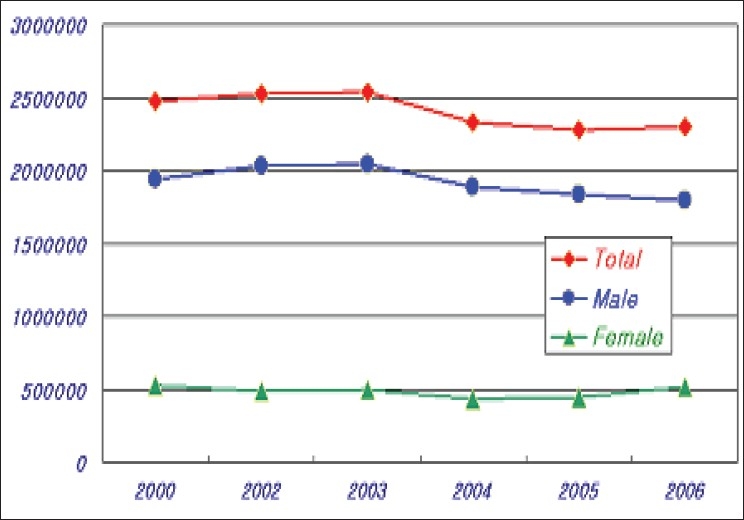
The population's donation number

**Figure 3:**
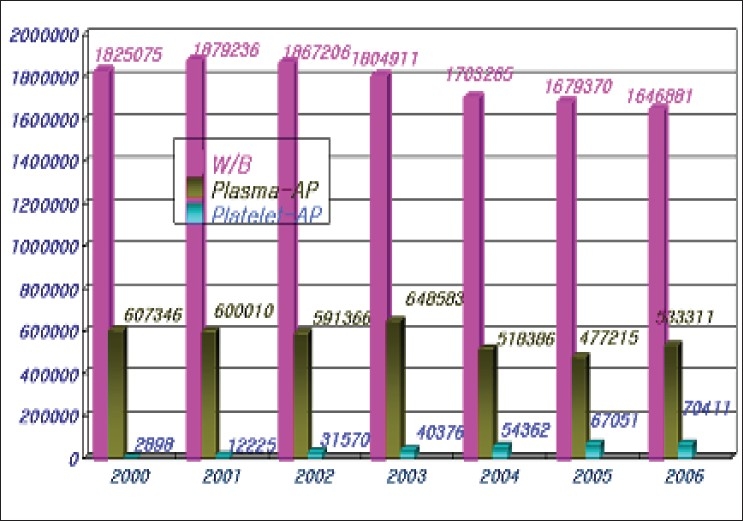
Types of blood donation

The average number of blood donation during the last three years was 2.3 million units. The KRC collected 98% of blood donated within Korea. All donors are voluntary-unpaid donors. The 7.6% of total blood donations decreased compared to the past years [Table 1, Figure 1]. In the last three years, an average of population's donation rate was 4.7%. The gender distribution of the donors is very uneven. Majority donors are male donors and female donors constituted only 20% of total blood donation. Teenagers and blood donors below 30 years of age constitute majority of blood donors from Korea. Young donors such as high school students easily participate in blood donation. However, it is not easy to retain them as regular donors [Table 2, Figure 2]. Whole blood composed 75% of total collected blood. Average amount of collected W/B during last three years was 1,67 million unit; 70% of which was 400 ml volume. Collection of single donor platelets has increased upto 127% in last three years [Table 3, Figure 3].

**Table 1:** Number of blood donation

	KRC	Medical private	Total
04	2,276,013 (97.9%)	49,095	2,325,108
05	2,223,636 (97.8%)	50,700	2,274,360
06	2,250,603 (97.8%)	51,026	2,301,629

**Table 2:** The population's donation rate

	Male	Female	Total
04	1,887,028	438,080	2,325,108
05	1,831,613	442,723	2,274,336
06	1,792,038	510,504	2,302,542

**Table 3:** Types of blood donation

	W/B	Plasma-AP	Platelet-AP	Total
04	1,703,265	518,386	54,362	2,276,013
05	1,679,370	477,215	67,051	2,223,636
06	1,646,881	533,311	70,411	2,250,603

**Figure 4:**
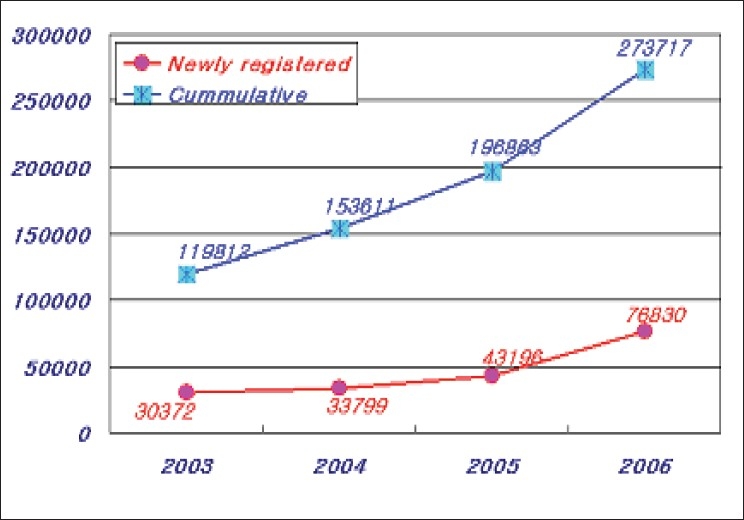
The number of registered donors

**Figure 5:**
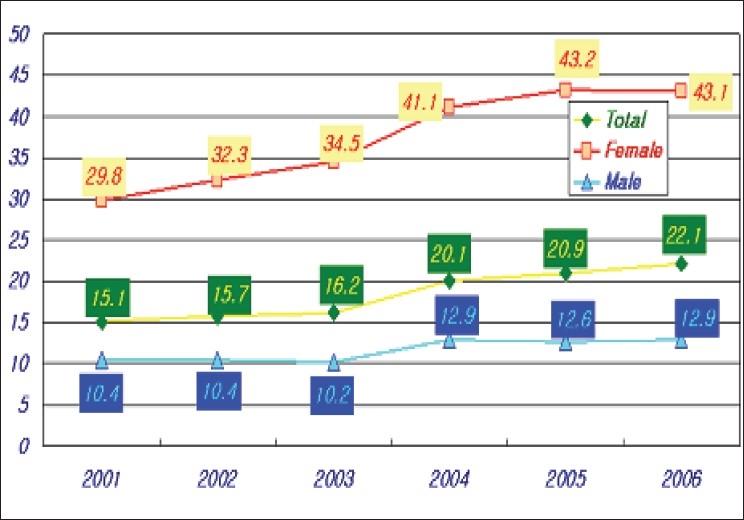
Donation deferral rate

**Figure 6:**
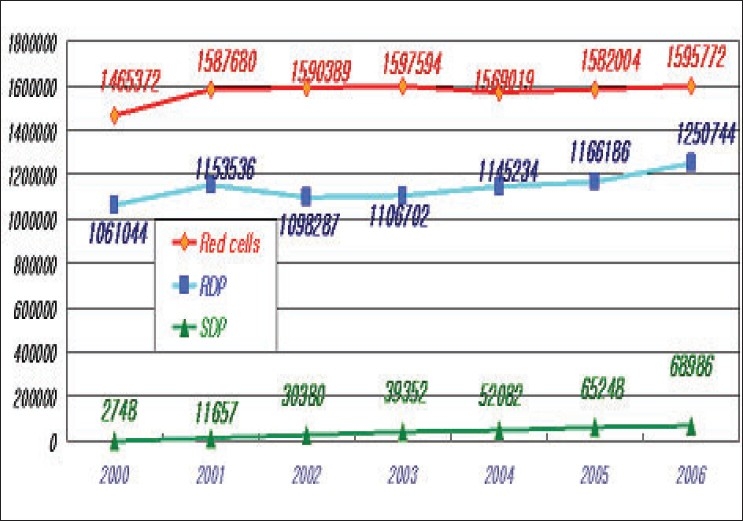
Blood components supplied to hospitals

The Korean Red Cross has started donor registry program since 2000. Currently, the total number of registered donors is about 3,40,000. These repeat blood donors donate blood three times in a year and 25% of total collected blood units are coming from these blood donors [Figure 4]. The deferral rate of donors was 22% in 2006 and it has been increasing since 2004. The KRC regulated stricter donor criteria and pre-donation counseling since late 2003 in accordance with National Blood Guidelines. The information related to malaria and drugs made deferral rate further increased [Figure 5]. The average amount of blood components supplied for transfusion during last three years was about 3.5 million units which was slightly increased compared to the previous years(3.43 million) [Table 4, Figure 6]. Compared to the increasing rate of Red blood cell supply, the supply of platelets including single donor platelets increased remarkably.

**Table 4:** Blood components supplied to hospital

	Transfusion	Source plasma	Total
04	3,456,491	1,504720	4,961,211
05	3,472,979	1,458,520	4,931,499
06	3,560,117	1,560,710	5,120,827

## Laboratory Tests

**Figure 7:**
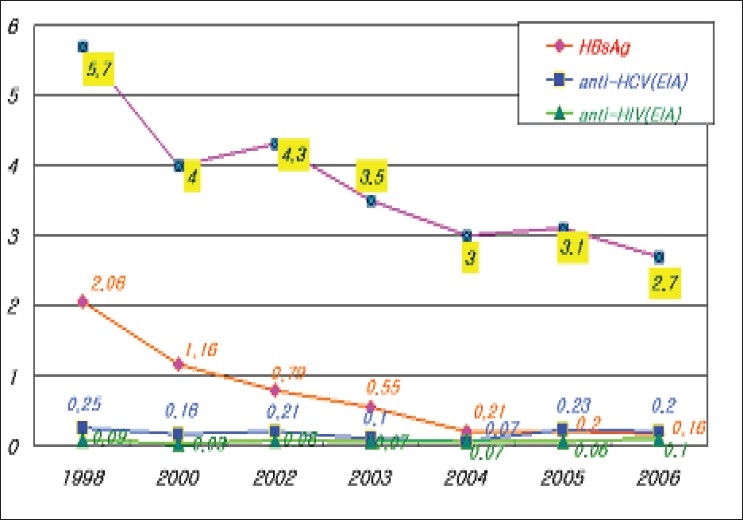
Positive rate of donor screening tests

Laboratory test method has been changed and developed to meet the needs of the times. Anti-malarial antibody test started in 2000. NAT for HIV and HCV was implemented in 2005. CLIA system for HBs Ag started in June this year. Preliminary HTLV test is planned to begin in December, 2007. Including high ALT value, abnormal blood screening rate was 2.7% in 2006. After the general implementation of pre-donation DDR screening in late 2003, positive rate of infectious markers decreased [Figure 7].

## Donation Deferral Registry (DDR)

**Figure 8:**
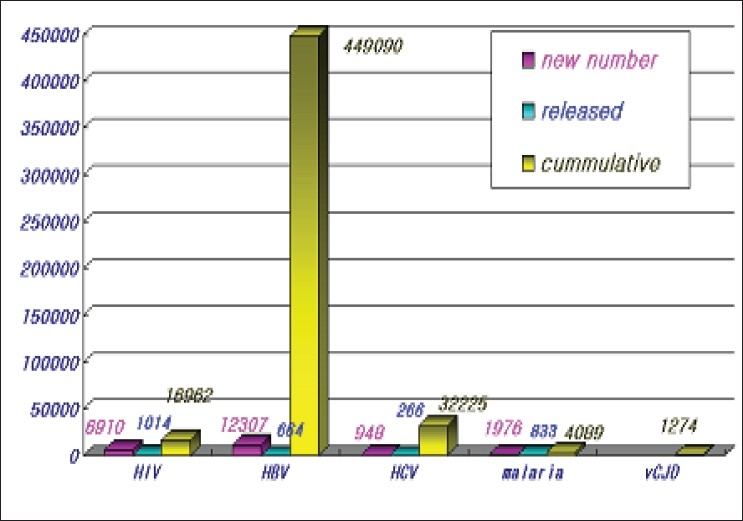
Donation deferral registry

DDR is data-base containing deferral donor's information. Unlike DDR, High Risk Group, so called HRG related to only HIV information was available in past years. In late 2003, DDR can be contained additional donor information such as positive results in HCV, HBV test and RNA test. Those who had history of diagnostic malaria, Hx of risk on drug also can be recorded. People having abnormal results are refrained from blood donations temporarily or permanently. The deferral donor information is used for pre-donation screening at all donation sites using mobile-phone or PC. Information of 500,000 donors are registered in this program until the end of 2006. The highest number of banned donors is related to HBV [Figure 8].

## Discard Rate

The overall discard rate in 2006 was 3.2% and it has been decreasing since 2000. Through DDR application, number of positive rates in laboratory tests was reduced. The shortened blood reserve resulted in dropping the discard rate of unused normal blood components [Table 5].

**Table 5:** Discard rate of blood components

	Red Cell	Platelets	SDP	Total discard
04	4.9	7.0	2.1	5.1
05	5.0	6.6	2.7	4.6
06	3.2	3.2	2.1	2.9

## Current Issues During Three Years

In Korean Red Cross blood service, there were two critical issues. First is ‘Security’ of blood safety. In late 2003, in 4 cases HIV infected blood was transfused to recipients during the window period. And that time, some cases of distribution of unqualified blood products were disclosed. The government directed the KRC to investigate the KRC laboratory system. Having investigated, old instruments (poor laboratory infra), poor performance of staffs, and failure to follow SOP were related to the distribution of tainted blood products. Aftermath blood scandal, a Planning Committee at governmental level was established in 2004. The Committee recommended the measures securing blood safety and substantial blood supply. Donor registry program for secure safe donors, laboratory automation and consolidation, construction of look back system with sample storage program, implementation of NAT in 2005, development of integrated blood information management system, so called BIMS, and reinforcement of inspection system are included. Most of them have been completed in 2007.

After implementation of NAT for HCV and HIV, transfusion transmission of window period HIV and HCV has not been reported until now. Over the last two years, total 14 cases of window period infected donor were detected by NAT. Second issue is about dropping the number of blood donors. Inventory levels of blood reserve is not enough to supply to hospitals demands. Extending malarial risk areas narrows the scope of potential blood donors. Less number of blood donations during lean seasons is also a major cause of reduction in blood collection.

## Conclusion

Public needs for safe blood would be continued. More accurate and precise laboratory test must be done. There should be regular training to all staff which in turn increases overall productivity. And blood shortage, poor laboratory infrastructure even though world wide trend. Blood as a public resource must be spared and utilized in accordance with clear guidelines. To ensure substantial blood supply, the KRC will further focus on increasing registered donors. Multi-component collections by aphaeresis should be also considered. In conclusion, I would like to stress that one of the most important elements to successfully carry out blood programs is to have strong and confident support from the public and the Government for the entire of KRC blood management.
